# Heat shock transcription factor 1 regulates exercise‐induced myocardial angiogenesis after pressure overload via HIF‐1α/VEGF pathway

**DOI:** 10.1111/jcmm.14872

**Published:** 2020-01-12

**Authors:** Xu Tian, Ning Zhou, Jie Yuan, Le Lu, Qi Zhang, Minmin Wei, Yunzeng Zou, Lingyan Yuan

**Affiliations:** ^1^ Department of Kinesiology Institute of Physical Education Shanghai Normal University Shanghai China; ^2^ Section of Cardiology Tongji Hospital, Tongji Medical College Huazhong University of Science and Technology Wuhan China; ^3^ Shanghai Institute of Cardiovascular Diseases Zhongshan Hospital and Institutes of Biological Science Fudan University Shanghai China

**Keywords:** cardiac angiogenesis, exercise training, HSF1, pressure overload

## Abstract

Exercise training is believed to have a positive effect on cardiac hypertrophy after hypertension. However, its mechanism is still not fully understood. Herein, our findings suggest that heat shock transcription factor 1 (HSF1) improves exercise‐initiated myocardial angiogenesis after pressure overload. A sustained narrowing of the diagonal aorta (TAC) and moderately‐ intense exercise training protocol were imposed on HSF1 heterozygote (KO) and their littermate wild‐type (WT) male mice. After two months, the cardiac function was assessed using the adaptive responses to exercise training, or TAC, or both of them such as catheterization and echocardiography. The HE stains assessed the area of myocyte cross‐sectional. The Western blot and real‐time PCR measured the levels of expression for heat shock factor 1 (HSF1), vascular endothelial growth factor (VEGF) and hypoxia inducible factor‐1 alpha (HIF‐1α) in cardiac tissues. The anti‐CD31 antibody immunohistochemical staining was done to examine how exercise training influenced cardiac ontogeny. The outcome illustrated that exercise training significantly improved the cardiac ontogeny in TAC mice, which was convoyed by elevated levels of expression for VEGF and HIF‐1α and preserved the heart microvascular density. More importantly, HSF1 deficiency impaired these effects induced by exercise training in TAC mice. In conclusion, exercise training encourages cardiac ontogeny by means of HSF1 activation and successive HIF‐1α/VEGF up‐regulation in endothelial cells during continued pressure overload.

## 1 INTRODUCTION

Hypertension contributes to the morbidity and mortality associated with cardiovascular diseases and has been a major concern for decades.^1^ Hypertension‐induced cardiac hypertrophy displays a compensatory adaptation of the ventricular function in the early stages followed by myocardial fibrosis and apoptosis in the later stage, and it eventually leads to heart failure.^2^, ^3^ It has been widely reported that physical exercise can effectively improve cardiovascular diseases, including the reduction in the onset of pathological myocardial remodelling and systematic lowering of blood pressure in hypertension.^4^, ^5^ However, the exact molecular mechanisms of this exercise‐mediated cardioprotection are not completely known. It is worth noting that inadequate blood supply enhances the rapid transition from compensatory cardiac hypertrophy to heart failure.^6^, ^7^ Hence, improving myocardial angiogenesis is essential in maintaining myocardial systolic function in response to sustained pressure overload. Recent studies have shown that exercise training can promote the endothelial progenitor cells proliferation and inhibit the sparsity of myocardial microvessels in myocardial infarction mice.^8^, ^9^ Thereby, we suspect that exercise training‐induced myocardial angiogenesis may be essential in maintaining the systolic function in response to sustained pressure overload.

HSF1 is a transcription factor of heat shock stress‐associated genes located in the cytoplasm as an inactive monomer and regulates the expression of heat shock protein genes after activation because of temperature stress.^10^, ^11^ Prior studies showed that HSF1 has a cardiac protective role as it prevents myocardial fibrosis and pathological cardiac hypertrophy during ischaemia injury and pressure overload.^12^, ^13^, ^14^ Recent studies suggest that activation of HSF1 may also stimulate the process of angiogenesis thereby reducing cardiac hypertrophy. For instance, in the chronic pressure‐overload mouse model, HSF1 deficiency inhibits angiogenesis and promotes the transition from pressure overload–induced cardiac hypertrophy to heart failure, which is ascribed to the inhibition of the HIF‐1α pathway.^15^, ^16^ In the xenograft model, down‐regulation of nuclear HSF1 inhibits the expression of HIF‐1α, thereby inhibiting tumour angiogenesis and delaying the development of cancer in patients with lung cancer.^17^ We previously found that HSF1 has a pivotal function in the adaptive mechanism of exercise‐induced physiological cardiac hypertrophy and pressure overload–induced pathological cardiac hypertrophy.^12^ Given that accumulating evidence illustrates that HSF1 and its target HIF‐1α/VEGF signalling pathway play a crucial role in pressure heart failure and pressure overload–induced pathological cardiac hypertrophy, we have been suggested that interaction between HSF1/HSPs and its targeting HIF‐1α/VEGF signalling pathway might be strongly linked to the central mechanisms of exercise‐mediated cardioprotection after hypertension.

In the present study, by using transverse aortic constriction (TAC) in HSF1 KO mice, together with an 8‐wk treadmill running training programme, we examined the HSF‐1/HSPs and HIF‐1α/VEGF gene expression and angiogenesis in TAC mice heart following exercise training. These results show an important effect of exercise training on cardiac hypertrophy and hypertension and offer a therapeutic option of inhibiting pathological cardiac remodelling and improvement of cardiac function after hypertension.

## 2 METHODS AND MATERIALS

### 2.1 Animal characteristic

In the study, 140 adult male C57BL/6 mice (weighing 22‐24 g, 6 weeks of age) purchased from Shanghai Laboratory Animal Center (Chinese Academy, Shanghai, China). HSF1‐deficient heterozygote (KO) mice were generated as reported previously.^18^ All animal experiments were approved by the Animal Care and Use Committee of Fudan University and complied with the with ‘Guide for the Care and Use of Laboratory Animals (the Guide, NRC 2011)'. The experiment was carried out in two parts, in brief, mice were randomly allocated into 4 groups (20 mice per group) including wild‐type sham group (WT‐sham), wild‐type ET group (WT‐ET), wild‐type TAC group (WT‐TAC) and wild‐type TAC + exercised group (WT‐TAC + E) in part one, and we designed three groups including HSF1 knockout sham group (HSF1KO‐sham), HSF1 knockout TAC group (HSF1KO‐TAC) and HSF1 knockout TAC + exercised group (HSF1KO‐TAC + ET) in part two. The mice were reared in standard cages supplied with water ad libitum and food at controlled humidity and temperature with a 12:12 hours light–dark cycle.

### 2.2 Pressure‐overload model and experimental protocol

A chronic pressure‐overload model was established by the TAC or sham operation according to previous studies.^14^, ^18^ In summary, mice were anaesthetized by ketamine (25 mg/kg) injection and then injected with 2% isoflurane intranasally to maintain anaesthesia during surgery. Then, the mouse precordial chest was opened between the 2nd and 3th rib to access the heart after ventilation and intubation. The descending aorta was identified and constricted by a 6‐0 silk suture tied snugly around both the aorta and a blunted 27‐gauge needle. After ligation, the needle was immediately removed, after which the skin and chest were closed. In contrast, the sham‐operated mice underwent a similar procedure without constriction.

The mice underwent a moderate‐intense exercise training protocol (approximately 60% of their maximal aerobic velocity),^19^ which was performed in on a motor‐driven treadmill (Hangzhou, China) for 8 weeks. The training schedule for the initial two weeks comprised 0% grade, 30 min/day at 11 m/min, and the intensity and duration were increased each day to achieve a training of 60 min at 13 m/min, 0% grade. Subsequent training was performed at the moderate level in each training period (5 days per week). All post‐training sessions were carried out 2 days after the last exercise training session to prevent acute effects of exercise. The other four groups of mice remained at sedentary during the training period.

### 2.3 Haemodynamic measurements and Echocardiography

Echocardiography and invasive haemodynamic measurement were carried out on animal‐specific instrument (VisualSonics Vevo770) at pre‐operation and 2 days after the last exercise bout. Briefly, animals were anaesthetized by 2% isoflurane, and M‐mode images of the left ventricle (LV) were acquired. Cardiac function and structure features such as LV posterior wall end‐diastolic thickness (LVPWd), fractional shortening (FS), ejection fraction (EF), LV end‐systolic volume (LVESV), LV end‐diastolic volume (LVEDV), LV internal dimension in systole (LVIDs) and LV internal dimension in diastole (LVIDd) were recorded as reported in a previous study.^16^, ^18^ Each measurement was presented as an average of five consecutive cardiac cycles, which were recorded by 3 experienced technicians who were blinded to the mice group allocation. The haemodynamic examination was carried out using a 1.4‐F pressure catheter (SPR 671, Millar Instruments) advanced into the aorta to reach the LV via the right common carotid artery. This procedure was performed under anaesthesia with 2% isoflurane.^15^ The Powerlab system was connected to the transducer to record the cardiac haemodynamic and morphological features such as the maximal contraction and relaxation velocity (max d*p*/d*t* and min d*p*/d*t*).

### 2.4 Histological analysis

The heart tissues were obtained from mice two days after the last exercise training. The LV, tissue was excised from the heart and then snap‐frozen in liquid nitrogen. The samples were divided for mRNA measurement and protein measurement. For staining experiments, the tissue wa fixed in 4% formalin dissolved in 0.1 M PBS (pH 7.4) for 24 hours. Thereafter, the samples were embedded in paraffin, and transversely cut into 5 μm sections onto slides in preparation for histological staining. The cross‐sectional area (CSA) of myocyte was determined by a quantitative digital image analysis (Image‐Pro Plus 6.0) using images that were acquired from HE‐stained sections. In each mice, >100 myocytes were counted per group. Some sections were treated with anti‐CD31 antibody (1:100; A Bclonal Biotech). This was followed by addition of 0.3% hydrogen peroxide solution to inhibit endogenous peroxidase activity. Tissue sections were examined under a light microscope (Nikon). Capillary density was quantitatively measured under a ×400 magnification microscope for five microscopic fields randomly chosen. The ratio of CD31 positive cell to all area was calculated by Image analysis software, and results were presented as average.

### 2.5 Real‐time PCR

The TRIzol reagent (Invitrogen, catalog 15596‐018, USA) was used to isolate total RNA from the LV tissues. About 1 mg RNA was used to prepare cDNA with the TOYOBO ReverTra Ace‐α‐RT‐PCR kit following the manufacturer`s guidelines. RT‐PCR was carried out on a Bio‐Rad IQ5 multicolour detection system using the cDNA sample. The relative mRNA expression was calculated based on the comparative CT method. All PCRs were run at least in triplicates, and the mRNA level of GAPDH was used as the internal control. The CT values were analysed by 2−ΔΔ*C*_t_ method. The genes for GAPDH, VEGF, HIF‐1α), HSP70, HSF1, skeletal βMHC, BNP and ANP were amplified the primers (Table S2).

### 2.6 Western blotting

The protein expression was determined based on the previously used protocol.^20^ Briefly, ventricular samples were lysed and then subjected to the BCA assay (Sigma‐Aldrich). The protein samples were loaded into the SDS‐page at equal concentrations and separated by electrophoresis. They were then transferred to nitrocellulose membrane (Millipore). GAPDH served as the control of total protein expression. The blots were examined and analysed Image J program.

### 2.7 Statistical analysis

The 2‐tailed Student's *t* test was used to compare two groups while multiple groups were compared by one‐way or two‐way ANOVA followed by LSD procedure. Data are presented as mean ± SEM *P* < .05 was considered statistically significant.

## 3 RESULTS

### 3.1 Exercise training protects against TAC‐induced pathological remodelling

**Figure 1:**
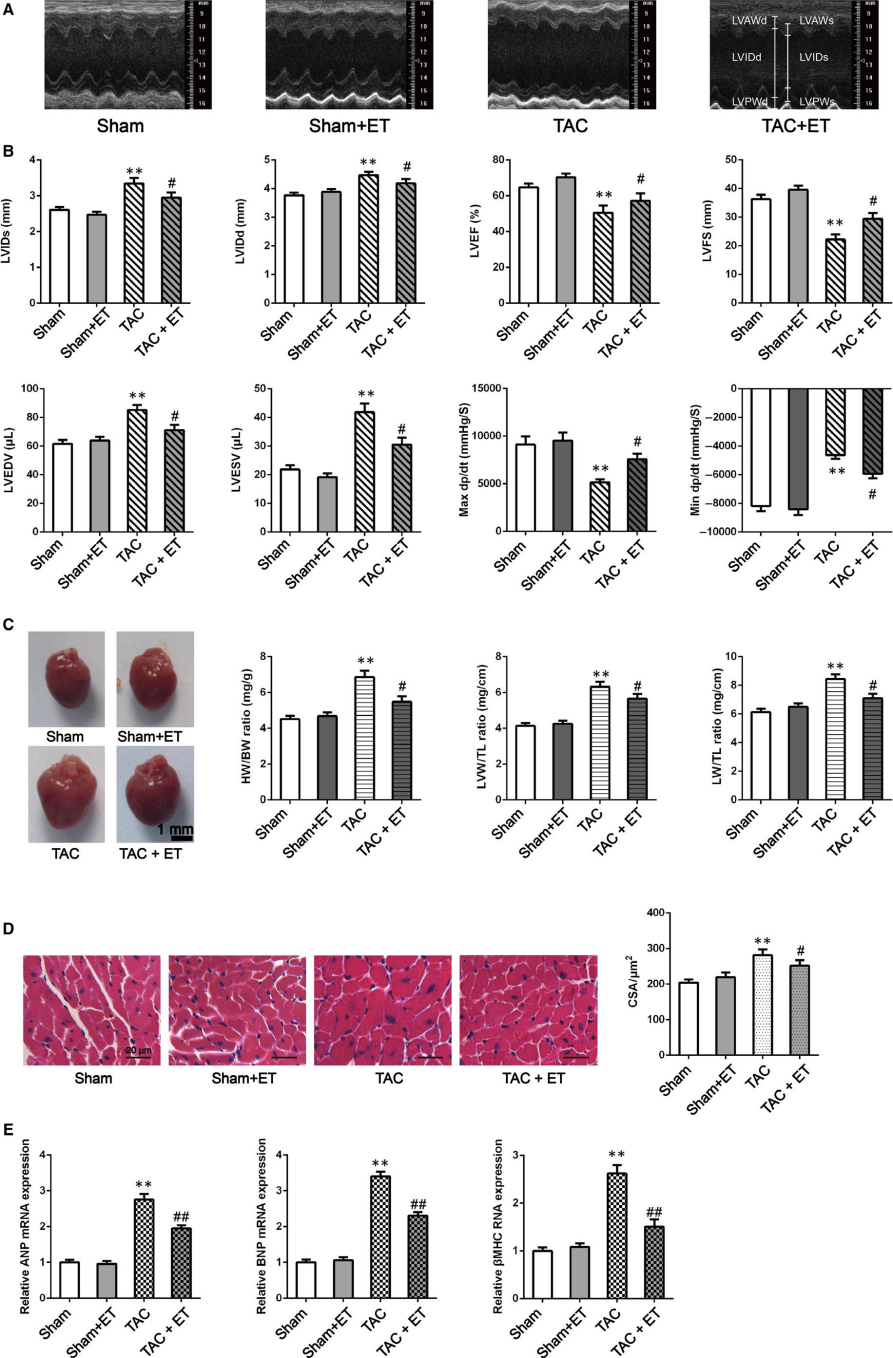
Exercise training improved cardiac pathological remodelling in TAC mice. A, B, Representative M‐mode echocardiography, echocardiographic analysis and Hemodynamic analysis in the Sham, Sham + ET, TAC and TAC + ET groups at two days after the last exercise bout. N = 6‐7 per experimental group. C, Heart morphology, HW/BW, LVW/TL and LW/TL. Representative global heart photographs. scale bar: 1 mm. N = 8 independent experiments (D) H‐E stained LV sections of mice. scale bar: 20 μm. CSA, cross‐sectional area of the cardiomyocyte. N = 6‐7 in each group. D, The expressions of ANP, BNP and βMHC were measured by QRT‐PCR. The mRNA expression of ANP/BNP/βMHC was quantified as the ratio of ANP/BNP/βMHC to GAPDH and expressed as 100% of Sham. All data shown are means ± SEM. *P < .05/**P < .01 vs Sham group; #*P* < .05/##*P* < .01 vs TAC group

To measure cardiac morphology and function of mice, M‐mode echocardiography and haemodynamic measurements were assessed in TAC, Sham, ET and TAC + ET groups 2 days after the last exercise bout. Figure 1A, which shows the representative M‐mode echocardiography in the four groups, indicates that cardiac dysfunction with LV dilatation occurred in TAC mice and exercise training suppressed the occurrence of TAC‐induced pathological remodelling in mice. Figure 1B shows that FS and EF in TAC group markedly reduced relative to that of Sham group, and this was accompanied by elevated LVEDV, LVESV, LVIDs and LVIDd, as well as decreased Min d*P*/d*t* and Max d*P*/d*t*, thereby indicating cardiac dysfunction in the TAC group. After exercise training, FS and EF notably recovered, and LVEDV and LVESV, as well as LVIDd, LVIDs, Min d*P*/d*t* and Max d*P*/d*t*, also improved in the TAC + ET group (Figure 1A). Besides, the results also indicated that LVPWd thickened significantly in TAC mice and exercise training suppressed the occurrence of TAC‐induced cardiac wall thickness increasing in mice. (See Table S1).

To further evaluate the link between exercise training and TAC‐related cardiac hypertrophy, we assessed the cardiac morphological parameters and the genes expression related to cardiac hypertrophy in the mice. As shown in Figure 1C, lung weight/tibia length ratio (LW/TL), LV weight/tibia length ratio (LVW/TL), heart weight/body weight ratio (HW/BW) and heart size, in the TAC group were markedly higher relative to those in the Sham group. HE staining results displayed an obviously enlarged cross‐sectional area (CSA) of the cardiac myocytes in the TAC mice than the Sham mice (Figure 1D). QRT‐PCR analysis for the expressions of faetal genes such as ANP, BNP and βMHC in the LV revealed an obvious advanced response to the pressure overload (Figure 1E). In contrast, exercise training attenuated changes in cardiac hypertrophy in TAC and TAC + ET groups (Figure 1C–E).

The heart rate was not significantly different amongst the four groups (see Table S1). Our results indicated that exercise training markedly improved pathological remodelling and cardiac function in TAC mice.

### 3.2 Exercise training restores cardiac microvessel function in TAC mice

**Figure 2:**
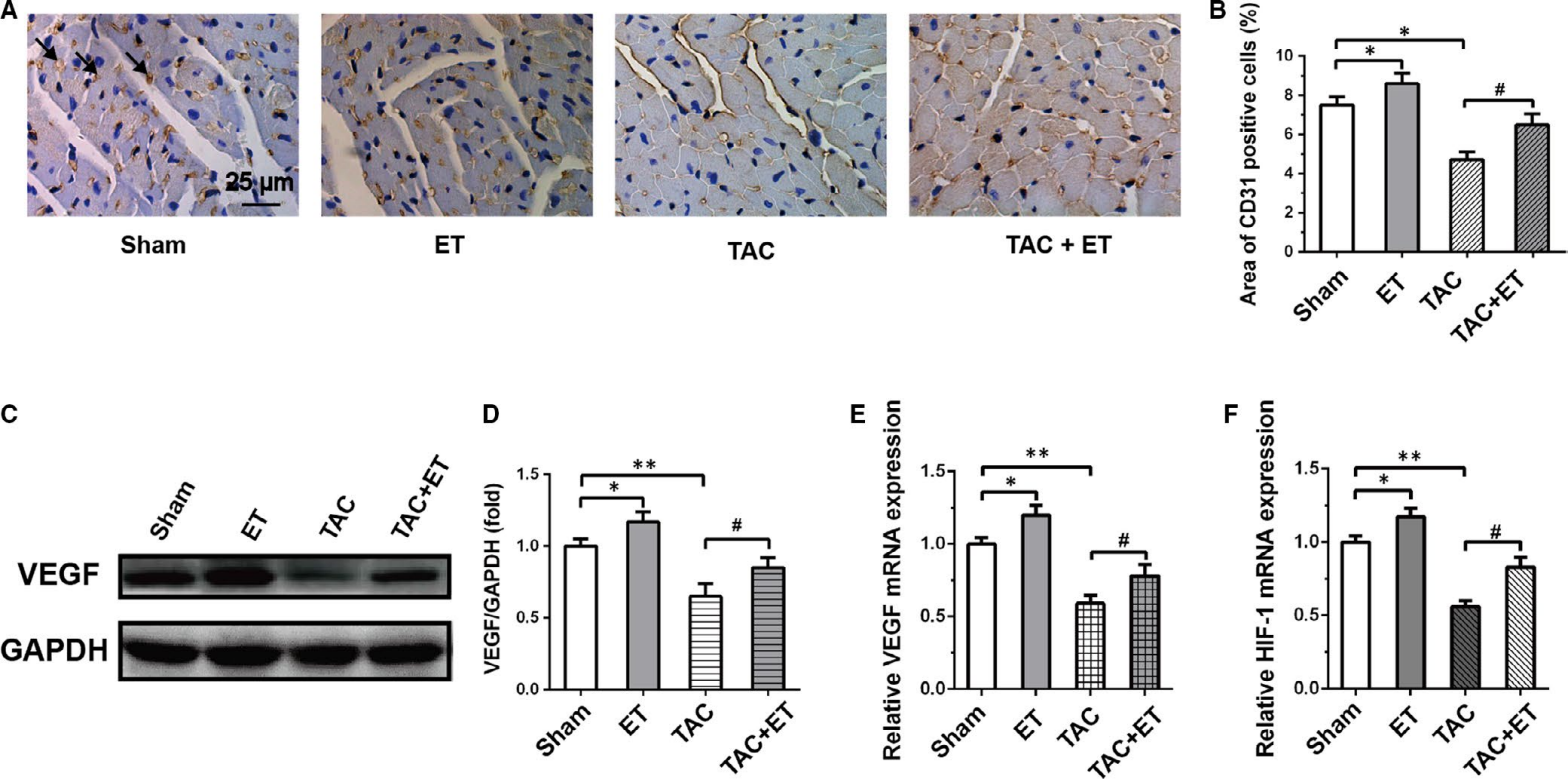
Exercise training alleviated cardiac microvessel impairment in TAC mice. A, Representative photographs of immunohistochemistry staining of the endothelial marker CD31 in myocardium sections (indicated by black arrows). scale bar, 25 μm. B, Quantitation of CD31 expression level in the heart. n ≥ 5 per group. C, Representative immunoblots for VEGF in the heart. D, Quantitative analysis of cardiac expression of VEGF measured by Western blot. E, F, Quantitation of VEGF and HIF‐1α expression level in the heart measured by real‐time PCR. All data shown are means ± SEM. n = 3 per group. *P < .05/ **P < .01 vs Sham group; #*P* < .05/##*P* < .01 vs TAC group. Immunohistochemistry staining of the endothelial marker CD31 in myocardium sections (indicated by black arrows)

The effect of TAC and exercise training on cardiac angiogenesis was examined by immunohistochemistry using anti‐CD31 antibody. Figure 2A,2, reveals that CD31 expression, a tissue microvessel density marker, was markedly increased in the ET group and obviously decreased in the TAC group. In the TAC + ET group, CD31 level was markedly raised, indicating that exercise training can promote myocardial angiogenesis and restored the reduction in microvessel density induced by TAC. It was found that the protein level of VEGF in the ET group were markedly up‐regulated relative to the Sham group, while in the TAC group VEGF protein expression level was obviously down‐regulated compared with those in the Sham group; exercise training remarkably increased the VEGF expression levels in TAC mice (Figure 2C,2). Moreover, the VEGF mRNA level of ET group was markedly higher but reduced in TAC group relative to Sham group, while VEGF mRNA level of TAC + ET group was markedly increased (Figure 2E). In addition, real‐time PCR analysis revealed that HIF‐1α, a key regulator of angiogenesis factor production, was markedly increased in ET group and significantly down‐regulated in the TAC group relative to the Sham group. Conversely, the exercise training produced a notable up‐regulation in the mRNA level of HIF‐1α in TAC + ET mice by comparison with TAC mice (Figure 2F). These results suggest that exercise training significantly promoted myocardial angiogenesis of normal mice and reversed TAC‐induced down‐regulation of VEGF and HIF‐1α expression levels.

We also found that cardiac HIF‐1α/VEGF pathway was lower in TAC group relative to Sham group and exercise training‐induced cardiac HIF‐1α/VEGF signalling.

### 3.3 Exercise training up‐regulated the expressions of HSF1 and Hsp70 in the cardiomyocytes of TAC mice

**Figure 3:**
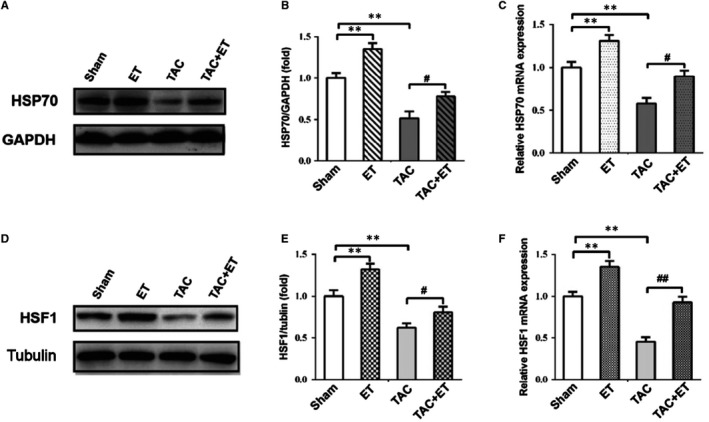
Effects of TAC and exercise training on the expression of HSF1 and Hsp70 in the hearts. A, Representative immunoblots for HSF1 in the heart. B, Quantitative analysis of cardiac expression of HSF1 measured by Western blot. C, Quantitation of HSF1 expression level in the heart measured by real‐time PCR. D, Representative immunoblots for Hsp70 in the heart. E, Quantitative analysis of cardiac expression of Hsp70 measured by Western blot. F, Quantitation of Hsp70 expression level in the heart measured by real‐time PCR. All data shown are means ± SEM. n = 3 per group. *P < .05/**P < .01 vs. Sham group; #*P* < .05/##*P* < .01 vs. TAC group

To assess the impact of TAC and exercise training on the level of HSF1 and Hsp70, Western blotting and QRT‐PCR was performed. As shown in Figure 3A,3, Western blotting analysis revealed that HSF1, a key regulator of Hsps expression, was significantly increased in ET group and obviously down‐regulated in TAC group relative to Sham group, the exercise training displayed a marked rise in the protein level of HSF1 in TAC + ET mice compared with TAC mice. Moreover, QRT‐PCR analysis showed that HSF1 mRNA levels in the ET group was markedly elevated and in the TAC group, it was apparently decreased relative to that in the Sham group respectively, whereas the HSF1 mRNA expression in TAC + ET group was markedly increased compared to the TAC mice (Figure 3C).

The inducible 70‐kD HSP family member Hsp70 is the most famous heat shock protein and is believed to be involved in myocardial protection during cardiovascular stress. The Western blotting analysis revealed that protein levels of Hsp70 was obviously up‐regulated in ET group and significantly down‐regulated in the TAC group compared with the Sham group (Figure 3D,3). On the contrary, these decreases induced by pressure overload were obviously improved by exercise training treatment (Figure 3D,3). QRT‐PCR analysis also confirmed that the level of Hsp70 were markedly increased in ET mice and down‐regulated in TAC mice relative to Sham mice; Compared with TAC mice, the above decrease was up‐regulated in TAC + ET mice (Figure 3F). These results indicate that exercise training can improve the heat stress response in TAC mice by modulating the expression of HSF1 and Hsp70.

### 3.4 HSF1 deficiency compromises the cardiac adaptive response of TAC mice to exercise training

**Figure 4:**
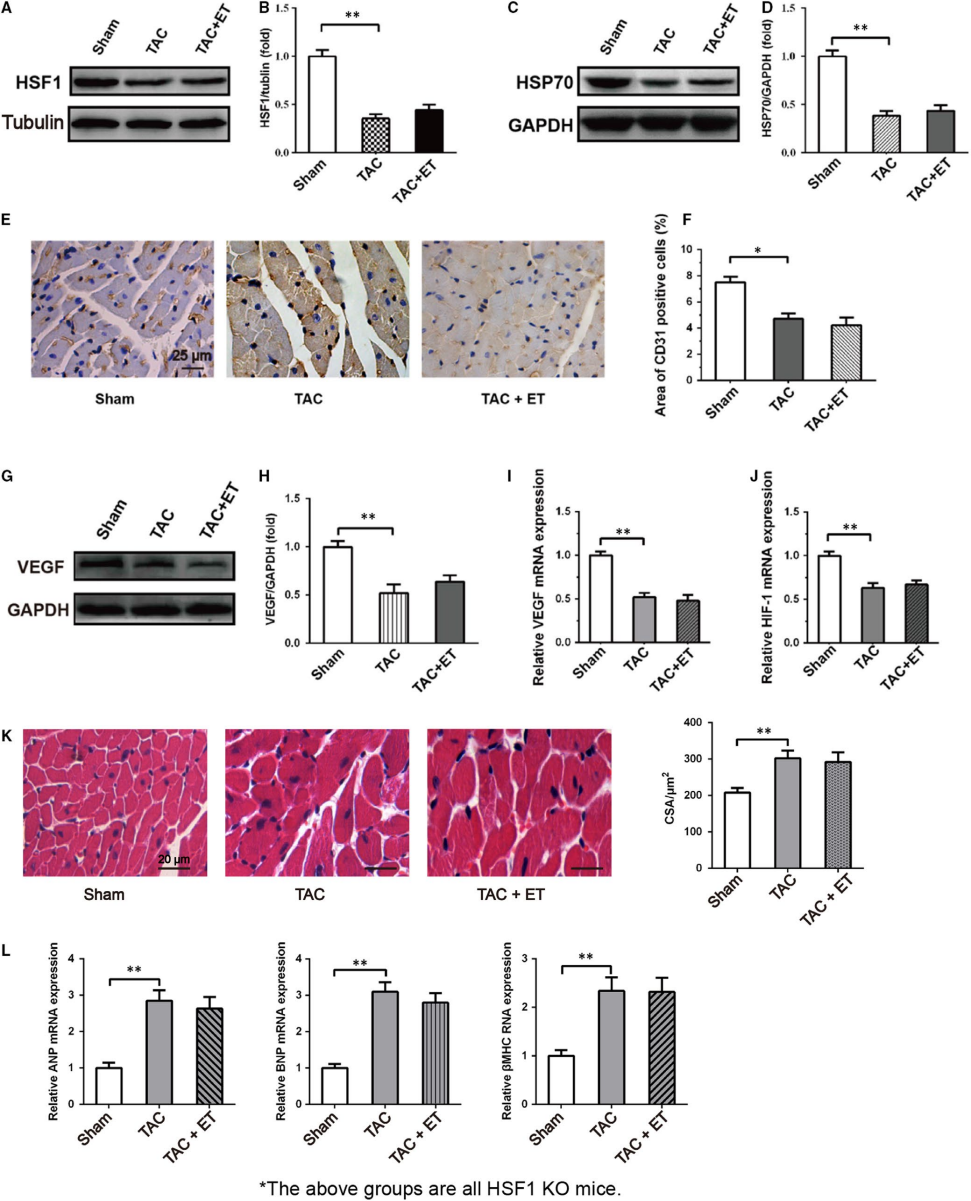
HSF1 deficiency impaired the beneficial effects induced by exercise training in TAC mice. A, Representative immunoblots for HSF1 in the heart. B, Quantitative analysis of cardiac expression of HSF1 measured by Western blot. C, Representative immunoblots for Hsp70 in the heart. D, Quantitative analysis of cardiac expression of Hsp70 measured by Western blot. E, Representative photographs of sections of left ventricles with immunohistochemical staining with the anti‐CD31 antibody. scale bar, 50 μm. F, Quantitation of CD31 expression level in the heart. n ≥ 5 per group. G, Representative immunoblots for VEGF in the heart. H, Quantitative analysis of cardiac expression of VEGF measured by Western blot. I, J, Quantitation of VEGF and HIF‐1α expression level in the heart measured by real‐time PCR. All data shown are means ± SEM. n = 3 per group. K, H‐E stained LV sections of mice. scale bar: 20 μm. CSA, cross‐sectional area of the cardiomyocyte. N = 6‐7 in each group. L, The expressions of ANP, BNP and βMHC were measured by QRT‐PCR. The mRNA expression of ANP/BNP/βMHC was quantified as the ratio of ANP/BNP/βMHC to GAPDH and expressed as 100% of Sham.*P < .05/**P < .01 vs. Sham group; #*P* < .05/##*P* < .01 vs. TAC group

Experiments were conducted to explore whether HSF1 potentiates cardiac adaptive response of TAC mice to exercise training. We produced the same model as the wild mice in HSF1‐deficient heterozygote (HSF1±) mice and compared them to their wild‐type littermates. Western blot assay showed that the level of HSF1 in the heart of HSF1± mice decreased by about 55% compared with the wild‐type mice. (data not shown). The HSF1± and WT adult mice were distinctly healthy, and without any marked difference in HR, BW, HW, LVAWd, LVEF, LVEDV, LVESV, FS, LVIDd, LVIDs, Min d*P*/d*t* and Max d*P*/d*t* amongst the mice before TAC (data not shown). Consistent with previous data (Experiment 1; for wild‐type mice), at 8 weeks after TAC, HW/BW, FS, LVEF, Max d*P*/d*t* and Min d*P*/d*t* were similarly declined as well as LVEDV, LVESV, LVIDd and LVIDs were similarly elevated in the HSF1KO‐TAC group relative to the HSF1KO‐Sham group mice (data not shown). Unlike the preserved function of wild‐type mice, the cardiac function of HSF1KO‐TAC mice was not enhanced after exercise training (In the HSF1KOTAC + ET group *vs* HSF1KO‐TAC group). In addition, QRT‐PCR and Western blot showed very consistent results that the level of HSF1 was conspicuously decreased in the heart of HSF1± mice by TAC, and this decrease was not improved by exercise training (Figure 4A‐D). Moreover, we also found that the expression of Hsp70 was obviously decreased in the heart of HSF1KO‐TAC mice, but this decrease wasn't attenuated in the heart of HSF1KO‐TAC + ET mice. In addition, HE staining results showed a distinctly enlarged cross‐sectional area (CSA) of the cardiac myocytes in the HSF1KO‐TAC group relative to the HSF1KO‐Sham group mice (Figure 4K). QRT‐PCR analysis for the expressions of faetal genes such as ANP, BNP and βMHC in the LV displayed an obvious advanced response to the pressure overload (Figure 4L). Unlike the wild‐type mice, the results of HSF1KO mice revealed that exercise training makes few changes in cardiac hypertrophy to the HSF1KO‐TAC + ET group relative to HSF1KO‐TAC group (Figure 4K‐L).

To further assess the role of HSF1 in promoting cardiac angiogenesis response of TAC mice to exercise training, immunohistochemical staining was carried out with anti‐CD31 antibody. Figure 4E,F shows that CD31 expression in the heart was markedly reduced in the HSF1KO‐TAC group as relative to the HSF1KO‐Sham group. There was no marked change in CD31 expression after exercise training in HSF1KO‐TAC mice (Figure 4E,F, HSF1KO‐TAC + ET group vs HSF1KO‐TAC group). Besides, Western blots and QRT‐PCR revealed that VEGF and HIF‐1α levels in the HSF1KO‐TAC group were remarkably reduced relative to those in the HSF1KO‐Sham group, and exercise training has little influence on VEGF expression levels in HSF1KO‐TAC mice (Figure 4G,H).

These results collectively suggest HSF1 has a protective role in the cardiac adaptive response of TAC mice to exercise training, further supporting a notion that HSF1‐dependent microvascular formation by cardiac endothelial cells is mediated by exercise‐regulated HIF‐1α protein.

## 4 DISCUSSION

**Figure 5:**
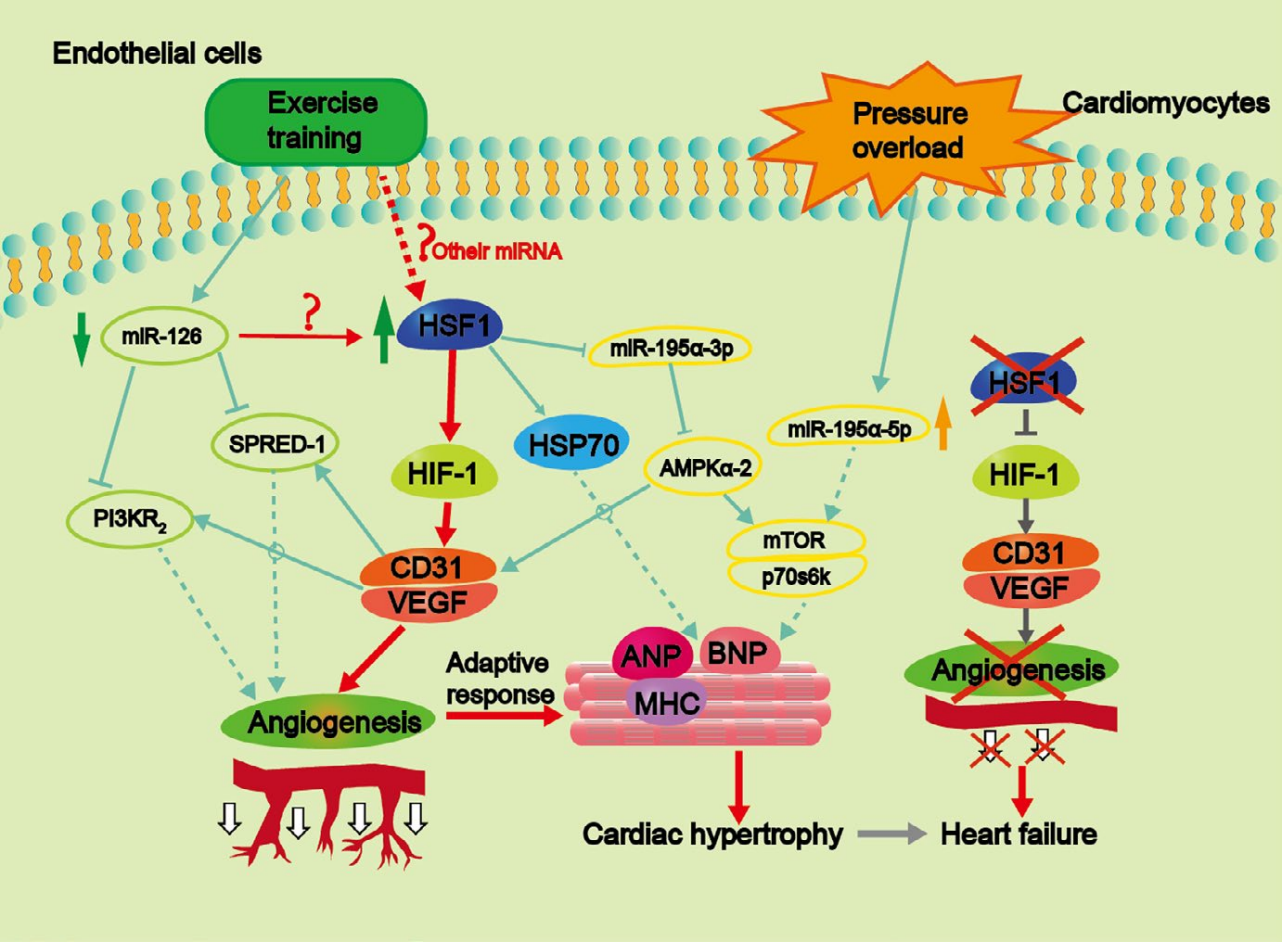
Schematic overview of the proposed mechanism: pressure overload induces the up‐regulation of miR‐195a‐5p in cardiomyocytes, which promotes cardiac hypertrophy via suppression of the cardiac metabolic pathway and up‐regulation of β‐MHC, BNP and ANP, and may finally lead to heart failure; In contrast to pressure overload, exercise training against pressure overload–induced cardiac dysfunction and hypertrophy via up‐regulation of HSF1 and promoting HIF‐1α/VEGF pathway (As displayed: Exercise has been pro can promote myocardial angiogenesis and prevent the transition from cardiac hypertrophy to heart failure via miR‐126/SPRED‐1 signalling pathway and miR‐126/PI3KR2 signalling pathway); HSF1 deficiency inhibits the expression of HIF‐1α and downstream VEGF, thereby lead to cardiac angiogenesis defect and impairs the cardiac adaptive response of TAC mice to exercise training

Here, we provide compelling evidence that (a) exercise training has a protective effect against TAC‐initiated pathological remodelling and cardiac ailment. (b) Exercise training reduces the cardiac microvascular disorder in TAC mice. (c) Exercise training up‐regulated the expression levels of Hsp70 and HSF1 in the cardiomyocytes of TAC mice. More importantly, we demonstrate the essential function of HSF1 in the signalling cascades, in which the physiological response to exercise training ameliorated the overgrowth responses and systolic disorder in WT mice which were subjected to excess pressure, while HSF1 deficiency abolished these actions. Therefore, we recommend a new molecular approach which illustrates that HSF1 and its target HIF‐1α/VEGF signalling pathway influences the exercise training‐activated cardioprotection against pressure overload–initiated cardiac hypertrophy (Figure 5).

Preceding studies reported that cardiovascular conditions including hypertension and heart failure are commonly linked to pathological hypertrophy induced by pressure overload.^2^, ^3^ Conversely, moderate exercise, which was earlier known for improving heart function in hypertension and heart failure, stimulates a multifaceted network of molecular processes that prevent stress‐related cardiomyopathy.^4^, ^5^ Nevertheless, the intracellular processes in which exercise training exhibits the cardioprotective actions are still to be revealed. Several studies have indicated the involvement of heat shock proteins (HSPs) and their transcription regular factors (HSFs).^12^, ^21^ The heat shock proteins, specifically the inducible isoform of the 70‐kD HSP family Hsp70, activate appropriate folding, cellular transference of synthesized proteins and avert the aggregation of proteins and denaturing destroyed proteins.^22^, ^23^ The Hsp70 synthesis is moderated at the level of transcription mainly by HSF1. The initiation of Hsp70 during exercise functionally restores the vital intracellular proteins destroyed during ischaemia‐related stress, which improves the normal heart function.^24^ Our previous study showed that wild‐type mice and HSF1 KO mice both developed similar extents of hypertrophy after four weeks of voluntary wheeling exercise, but HSF1 KO mice had notable reduced cardiac function after exercise training compared with wild‐type mice, suggesting how HSF1 influences the adaptive mechanisms of exercise‐related myocardial overgrowth.

In the present study, we adopted a long‐term chronic moderate exercise training scheme and investigated the exercise programme effects on the heart morphology and the role of pressure‐overload mice. Our data demonstrate that 2 months of aerobic exercise training partially averted cardiac ailment and the LV chamber dilation weakening. It also moderately averted pressure overload–initiated myocardial overgrowth. The outcome confirms one of our assumption that exercise training can improve the cardiac dysfunction associated with hypertrophy caused by pressure overload, which is in line with several previous studies.^25^, ^26^ Besides, in the Wild‐type mice, we found that the expressions of HSF1 and HSP70 were up‐regulated significantly both in the control group and pressure‐overload group after exercise training. On the basis of above observations, we infer that exercise exerts protection by preventing cardiac dysfunction due to pressure overload in mice by the up‐regulation of the HSF1 expressions and subsequently up‐regulation of the HSP70 expressions in the chronic phase of pathological myocardial hypertrophy.

Many studies confirmed that ontogeny influences the morbidity of hypertension and HF,^27^, ^28^ we investigated whether exercise training influences cardiac ontogeny in the normal heart and TAC‐induced pathological heart. To our knowledge, preceding studies did not provide sufficient evidence on the impact of exercise training on myocardial ontogeny in TAC‐induced cardiac remodelling exists. Nevertheless, a recent study reported that swimming exercises increase the expression levels of miRNA‐126, which moderates the target mRNAs (PI3KR2 and Spred‐1), thus contributing to the phenotypic features of cardiac ontogeny through MAPK and PI3K/Akt/eNOS signalling pathways of VEGF^29^ (Figure 5). From this study, we demonstrated that exercise training evidently increased cardiac angiogenesis both in normal mice and pressure‐overload mice, through the up‐regulation of VEGF and HIF‐1α expression levels and maintenance of the heart microvascular density. This revealed the potential effects of exercise training on averting TAC‐induced cardiac remodelling and improving cardiac ontogeny.

To further investigate the mechanism by which HSF1 improved the pressure‐overloaded heart during exercise, we designed a HSF1‐KO group and the wild‐type group in the second part of the experiment, and both groups of mice were exercised after TAC surgery, and changes in the cardiac function and angiogenesis indicators were observed after exercise. Our data illustrated that the expression of HSF1 and HSP70 were down‐regulated evidently in the heart of HSF1 KO mice in comparison with the levels in WT mice. Meanwhile, there were no evident differences in the indexes of cardiac structure and function, including echocardiogram parameters, haemodynamic parameters, Cardiac morphological parameters and the genes expression associated with cardiac hypertrophy, between the HSF1KO‐TAC group and HSF1KO‐TAC + ET group. In addition, we also found there were few changes in angiogenesis and genes expression of HSF1 and HSP70 between the HSF1KO‐TAC group and HSF1KO‐TAC + ET group. Altogether, the above data demonstrate that deficiency of HSF1 weakens the improvement of morbid cardiac remodelling and systolic disorder in the pressure‐overloaded heart induced by exercise training.

Despite these findings, the following limitations pertaining to this research we did not provide information on how exercise training moderates the expression of HSF1. The expression of HSF1 is affected by exercise training through regulating the expression of miR‐126 or other miRNAs (Figure 5). For example, DA Silva *et al* confirmed that miRNA126 is directly influenced during exercise training, thereby affecting myocardial angiogenesis.^29^ We hope that our findings lay the foundation for further detailed studies.

Briefly, this study clarifies the process by which exercise training improves TAC‐initiated cardiac remodelling and systolic ailment, and a crucial role of HSF1‐dependent lasting preservation of cardiac micro‐ontogeny in response to exercise training. We provide evidence that exercise training mainly stimulates HSF1 and its target HIF‐1α/VEGF signalling pathway axis thereby preventing the conversion of cardiac overgrowth into arrest. This current study reveals a possible biological target for treating cardiac hypertrophy related to hypertension.

## CONFLICT OF INTEREST

There is no evident competing interests amongst the authors.

## Data Availability

All data used to support the findings of this study are available from the corresponding authors upon request.
