# Supplementary material for: Heat shock transcription factor 1 regulates exercise‐induced myocardial angiogenesis after pressure overload via HIF‐1α/VEGF pathway
Source: J Cell Mol Med. 2020 Jan 12;24(3):2178–88. doi: 10.1111/jcmm.14872 (PMC7011135; doi:10.1111/jcmm.14872)
Supplement: Supplementary file 1 [file JCMM-24-2178-s001.docx]

**Supplementary Tables**

**Table 1.** Echocardiographic and hemodynamic analysis of mice 2 days after the last exercise training protocol (Experiment 1; Wild-type mice).

|  | Sham | Sham+ET | TAC | TAC+ET |
| --- | --- | --- | --- | --- |
| LVPWd (mm) | 0.65±0.04 | 0.69±0.05 | 0.88±0.12** | 0.80±0.10# |
| LVIDd (mm) | 3.77±0.09 | 3.88±0.10 | 4.47±0.12** | 4.19±0.14# |
| LVIDs (mm) | 2.61±0.08 | 2.47±0.09 | 3.35±0.15** | 2.95±0.14# |
| LVFS (%) | 36.3±1.53 | 39.6±1.40 | 22.2±1.8 ** | 29.4±2.1# |
| LVEF (%) | 64.7±2.2 | 70.3±2.1 | 50.6±3.9 ** | 57.2±4.1# |
| LVEDV (µL) | 61.7±2.5 | 63.9±2.5 | 85.2±3.5** | 71.17±3.7# |
| LVESV (µL) | 21.8±1.5 | 19.2±1.3 | 41.8±3.1** | 30.5±2.4# |
| Max dp/dt (mmHg/S) | 9120±856.5 | 9535±840.7 | 5153±306.3** | 7578±580.5## |
| Min dp/dt (mmHg/S) | -8178±380.2 | -8412±401.6 | -4627±256.5** | -5927±326.5## |
| HR (bpm) | 556.2±50.4 | 548.6±52.1 | 581.5±46.3 | 566.8±49.5 |

Sham indicates sham-operated mice; TAC and TAC+ET mice, respectively, receiving pressure overload by 8 weeks of TAC; Sham+ET and TAC+ET mice, respectively, receiving a moderate-intensity exercise after 3 days of TAC surgery. Note: LVPWd, left ventricular posterior wall end-diastolic thickness; EF, ejection fraction; LVEDV, left ventricular end-diastolic volume; LVESV, left ventricular end-systolic volume; FS, fractional shortening; LVIDd, left ventricular internal end-diastolic dim ensions; LVIDs, left ventricular internal dimension in systole; HR, heart rate; max dp/dt, maximal contraction velocity; min dp/dt, maximal relaxation velocity; *p<0.05, **p<0.01 *vs*. Sham mice. #p<0.05, ##p<0.01 *vs*. TAC mice.
