# Supplementary material for: Heat shock transcription factor 1 regulates exercise‐induced myocardial angiogenesis after pressure overload via HIF‐1α/VEGF pathway
Source: J Cell Mol Med. 2020 Jan 12;24(3):2178–88. doi: 10.1111/jcmm.14872 (PMC7011135; doi:10.1111/jcmm.14872)
Supplement: Supplementary file 2 [file JCMM-24-2178-s002.docx]

**Table 2.** A list of primers used in this study

| **Genes** | **Forward** **primer(5’-3’)** | **Reverse primer(5’-3’)** |
| --- | --- | --- |
| ANP | CCTGGACTGGGGAAGTCAAC | ATCTATCGGAGGGGTCCCAG |
| BNP | TGACGGGCTGAGGTTGTTTT | TGAGGTCAAAGGGCCTGGT |
| βMHC | TTGAGAATCCAAGGCTCAGC | TCCTCTGCTTCTTGTCCAGG |
| HSF1 | TCTCCTGTCCTGTGTGCCTAGC | CAGGTCAACTGCCTACACAGACC |
| HSP70 | GACGGGCTGAGGTTGTGT | AGGTCGAGGGTCTCCTCTGTC |
| VEGF | GAAGAAAGTGGTGCCATGGATAG | CCCATGAGTTCCATGCTCAGA |
| HIF-1α | CAACGTGGAAGGTGCTTCA | CGGCTCATAACCCATCAACT |
| GAPDH | GCAGTGGCAAAGTGGAGATT | TCTCCATGGTGGTGAAGACA |
